# Optical mapping discerns genome wide DNA methylation profiles

**DOI:** 10.1186/1471-2199-9-68

**Published:** 2008-07-30

**Authors:** Gene E Ananiev, Steve Goldstein, Rod Runnheim, Dan K Forrest, Shiguo Zhou, Konstantinos Potamousis, Chris P Churas, Veit Bergendahl, James A Thomson, David C Schwartz

**Affiliations:** 1 Department of Chemistry, Department of Genetics, Laboratory for Molecular and Computational Genomics, University of Wisconsin Biotechnology Center, University of Wisconsin-Madison, Madison WI 53706, USA; 2 Department of Anatomy, the Primate Center, University of Wisconsin Biotechnology Center, University of Wisconsin-Madison, Madison WI 53706, USA

## Abstract

**Background:**

Methylation of CpG dinucleotides is a fundamental mechanism of epigenetic regulation in eukaryotic genomes. Development of methods for rapid genome wide methylation profiling will greatly facilitate both hypothesis and discovery driven research in the field of epigenetics. In this regard, a single molecule approach to methylation profiling offers several unique advantages that include elimination of chemical DNA modification steps and PCR amplification.

**Results:**

A single molecule approach is presented for the discernment of methylation profiles, based on optical mapping. We report results from a series of pilot studies demonstrating the capabilities of optical mapping as a platform for methylation profiling of whole genomes. Optical mapping was used to discern the methylation profile from both an engineered and wild type *Escherichia coli*. Furthermore, the methylation status of selected *loci *within the genome of human embryonic stem cells was profiled using optical mapping.

**Conclusion:**

The optical mapping platform effectively detects DNA methylation patterns. Due to single molecule detection, optical mapping offers significant advantages over other technologies. This advantage stems from obviation of DNA modification steps, such as bisulfite treatment, and the ability of the platform to assay repeat dense regions within mammalian genomes inaccessible to techniques using array-hybridization technologies.

## Background

DNA methylation is a major epigenetic mechanism of gene regulation in higher eukaryotes. DNA methylation can be defined as the addition of a methyl group to the base of a nucleotide by DNA methyltransferases [1,2]. In prokaryotes, DNA methyltransferases operate in tandem with restriction enzymes as a part of a defense mechanism against invading viral DNA. Prokaryotic DNA methylation protects native DNA from cleavage by endogenous restriction enzymes, thus creating a defense mechanism against invading viral DNA [3]. In higher eukaryotes, DNA methylation acts to protect the genome, by silencing the expression of retroviruses [4]; however, it is gene regulation mediated by DNA methylation that is of principal interest.

Since the methylation of cytosine at position C5 in the context of cytosine guanine dinucleotides plays a significant biological role in higher eukaryotes, its detection forms the primary focus of our research. In higher eukaryotes, CpG dinucleotides are often clustered together forming CpG islands [5] that frequently coincide with upstream regulatory elements and promoters of genes. A consequence of methylation of upstream promoter regions is repression of targeted genes. Transcription repression occurs by either a direct obstruction of the major grove [6] or via a methylation binding protein [7] mechanism. In fact, methylation of CpG dinucleotides and CpG islands is the major mechanism for imprinting in eukaryotes. Methylation profiles are mediated by mechanisms associated with factors such as age, nutrition, disease, or mutational events that may induce pathogenic changes in gene expression. Both hypo- and hyper-methylation are known to play a role in the onset of oncogenic disease [8] by either activating oncogenes or by silencing tumor suppressor genes. Abnormal DNA methylation plays a role in a myriad of disease, with a notable example being schizophrenia [9,10].

The development of single molecule approaches for the discernment of genome wide methylation profiles that also obviate traditional chemical modification steps points the way towards creation of new high-throughput platforms. Current methods for analysis of DNA methylation require chemical treatment of DNA bases that do not readily query genomic repeat elements [11]. Bisulfite PCR [12] is the current "gold standard" method for assaying DNA methylation. During bisulfite treatment, cytosines are deaminated forming uracil while methylated cytosines remain unmodified. These changes can then be tested by PCR amplification of selected loci. To probe the methylation status of specific nucleotides, PCR products are cloned and sequenced. Post bisulfite sequence is compared to wild type, where unmethylated nucleotides appear as C to T point mutations. While bisulfite sequencing is an excellent technique for sampling specific loci with an underlying hypothesis in hand, the computational complexity of designing PCR primers and multiple requisite steps make it impractical for comprehensive genome analysis. Also due to inherent incompatibility of PCR analysis with regions of genomic repeats, it is difficult to use bisulfite PCR to analyze repeat regions of genomes, where high-resolution knowledge of methylation states may provide important biological insights.

Currently, methods combining bisulfite treatment of DNA with "BeadArray" hybridization are being used to assay the methylation state of selected loci within the human genome [13]. The BeadArray platform alleviates the inherent computational complexity of differential PCR primer design and obviates the need for hundreds of PCR reactions; however, such analysis does not interrogate many classes of genomic repeats. Another promising approach is the direct sequencing of bisulfite PC amplicons using 454 sequencing [14]. The 454 sequencing approach eliminates the need for PCR product cloning. Thousands of individual sequence reads are generated for each PCR product, compared to tens of reads generated using conventional bisulfite sequencing. The current methods for discernment of genomic methylation have been reviewed in great detail elsewhere [15,16].

In contrast to bisulfite PCR which is time consuming, costly and difficult to use on a large scale when coupled with PCR analysis, optical mapping offers many advantages for comprehensive methylation profiling. It is an established single molecule platform for investigation of whole genomes. Optical mapping has been used for the construction of complete physical maps of numerous bacterial, plant and human genomes [17-31] (for an in depth description of optical mapping see [32]).

**Figure 1:**
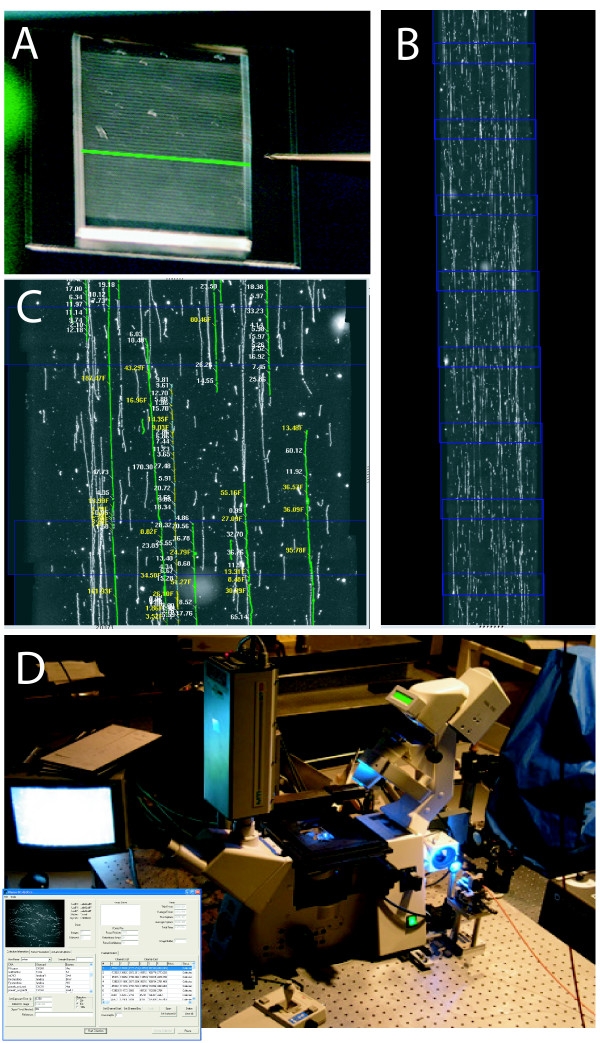
**Optical mapping system**. An overview of the optical mapping system. A; Large, genomic DNA molecules are elongated and arrayed as 15 mm long stripes onto positively charged surfaces using a microfluidic device; a green line depicts one channel (48 total). B; After restriction digestion and staining, an automated fluorescence microscope scanner ("Genome Zephyr"; [D]) serially acquires overlapping image frames along each of the 48 stripes laid down by the microfluidic device. ChannelCollect software flattens and overlaps images maintaining sub-pixel registration; ~8/170 overlapped image frames from one channel are shown. White "threads" are individual DNA molecules; blue boxes indicate each ~100 μm-wide frames. C; Machine vision (Pathfinder [32,33]) identifies molecules and constructs ordered restriction maps for each molecule; integrated fluorescence intensity measurements estimate mass of daughter restriction fragments in kilobasepairs. D; An optical mapping station known as Genome Zephyr; pictures show microscope, fiber-optic illumination, computer controlled stage. The insert shows the optical mapping software interface–ChannelCollect. The user identifies the start and end coordinates of the first and last channel on a surface. Further channels are identified by the software. Imaging and data processing are fully automated.

The OM system creates high-resolution physical maps of genomes based on ordered restriction maps of individual DNA molecules. Briefly, individual high molecular weight (~500 kb) DNA molecules are unraveled and arrayed upon positively charged glass surfaces using a microfluidic device [33]. After deposition, molecules are restriction digested, then stained with a fluorochrome dye and finally imaged by automated fluorescence microscopy (Figure 1). "Daughter" restriction fragments remain bound to the surface retaining original order allowing construction of *ordered *restriction maps from individual "parental" molecules. This step is accomplished through automated image processing and analysis of individual DNA molecules, which are converted into "molecular bar codes" based on the distances (kb) as measured by integrated fluorescence intensity between sites of restriction enzyme cleavage. The molecular bar code represents a unique identification "tag" for each individual molecule used by map alignment algorithms [34-36] and software for construction of whole genome physical maps.

Since optical mapping uses genomic DNA substrates, which retain inherent CpG methylation patterns, we reasoned that methylation sensitive restriction enzymes would profile such patterns of DNA modification on a whole genome basis. Because methyltransferases and restriction enzymes operate in tandem in bacteria, the *Escherichia coli *genome was used as a facile model system for methylation profiling by optical mapping. Since most restriction enzymes will not cleave methylated cognate sequences, modified sites are directly detected as missing restriction sites, thereby obviating chemical modification steps as part of this detection process. More importantly, by selecting appropriate restriction enzymes, we can bias the investigation of specific repeated elements within a mammalian genome, such as CpG islands or LINES [37], by keying an enzyme's cognate sequence towards cleavage within chosen elements.

As such, our detection strategies use: **(*i*) **solely methylation sensitive restriction enzymes, or **(*ii*) **a combination of methylation sensitive and insensitive enzymes within the same reaction. This enzyme combination, especially for mammalian genomes, produces interpretable bar codes through added flexibility in how single molecule restriction maps are created and then analyzed. Consider that "consensus" maps, or contigs–constructed from merging multiple, overlapping optical maps (single molecule)–are assessed for methylation states by comparison against a sequence based *in silico *map ("reference map"; a restriction map computationally constructed from sequence data). Consequently, scheme (*ii*) partitions genomic placement of such contigs from consideration of methylation status by anchoring contigs using only those restriction patterns created by the methylation insensitive enzyme. After contigs are placed against a reference map, methylation status is derived by comparing the nucleotide locations of methylation sensitive cleavage sites (cuts) within a contig to the corresponding *in silico *restriction map features of a reference genome. In other words, absence or presence of those restriction sites queried by the methylation-sensitive enzyme tabulates their modification state.

Strategy (*i*), described above, is applied for the detection of the methylation profile of both an engineered and a wild type *E. coli*. The results firmly validate this strategy for small genomes. We then employed strategy (*ii*), designed for analysis of complex genomes, for reporting CpG methylation of a mid passage (p44) human embryonic stem cell line H1. Generating only modest coverage of optical maps across the entire human genome, over 90 sites of DNA methylation were detected across Ch 9 in regions showing usable coverage by optical maps. These findings illustrate the feasibility of using a single molecule approach for *de novo *discovery of methylation patterns presented by complex genomes.

## Results

### Strategy for detection of methylation sites using only a methylation-sensitive enzyme

**Figure 2:**
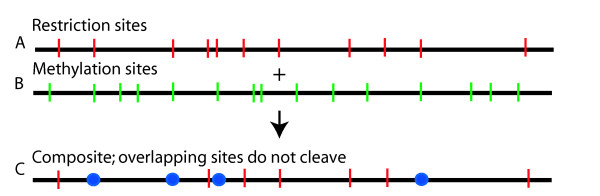
**Strategies for detection DNA methylation using restriction enzymes**. Detection of methylation patterns using a single methylation sensitive restriction enzyme. Knowledge of expected restriction sites, from an *in silico *map, in the absence of methylation, locates methylation sites revealed through actual restriction digestion. A; *In silico *restriction map; cleavage sites are shown as vertical red bars. B; A hypothetical distribution of DNA methylation sites; green bars. C; Composite restriction map (A + B) incorporating blocking effects from the overlap of restriction with methylation sites; location of missing restriction sites caused by overlap (blue circles).

**Figure 3:**
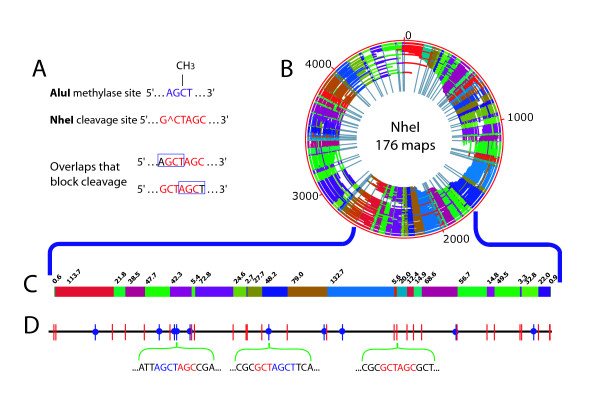
**Profiling of *E. coli *AluI methylation sites by NheI restriction mapping**. A; Overlaps between sites of NheI (red) restriction enzyme cleavage and AluI (blue) methylation blocking cleavage. B; NheI *de novo *optical map contig of AluI methylated *E. coli*, containing 176 maps. Outer red circle shows genome coordinates (kb) with internal arcs representing 176 maps; individual restriction fragments within each map are denoted by alternating colors; and grey radial lines demarcate restriction fragments within the contig map (next to the genome coordinates–red circle). The origin of the optical map does not coincide with the start of the published sequence, because the optical map was assembled *de novo*. C; An enlarged section (~960 kb) of the *de novo *NheI optical map contig. Colored blocks represent individual restriction fragments with their respective sizes (kb) marked above. D; Detailed comparison of the optical map shown in (C) against the corresponding *in silico *NheI map. NheI cleavage sites are shown as vertical bars; red bars show cleavage sites observed in the optical map. NheI sites overlapping with AluI methylation (absent in the optical map) are shown as blue vertical bars with a blue circle denoting blocking; below, the sequences around 3 NheI restriction sites are shown, two of which overlap AluI methylation sites.

We analyzed optical maps generated by a methylation sensitive restriction enzyme (NheI {G^CTAGC}) for revealing genome-wide patterns of DNA methylation (strategy (*i*)) engendered by AluI methylase (AGC^Me^T) on *E. coli *genomic DNA molecules. By engineering such methylation sites within a genome, in contrast to assessing naturally occurring methylation sites, we generate a list of known modified sites. Virtually all sites that block cleavage with NheI can be experimentally identified (see Figures 2 and 3).

Selection of appropriate methylase/restriction enzyme pairs is facilitated by the fact that most restriction enzymes do not cleave DNA if their recognition, or cognate sequence is methylated (in some cases, this is dependent on whether cytosines or adenines are modified). Given a list of genomic locations that are expected to be enzymatically methylated *in vitro*, calculated from sequence information, we then select a restriction enzyme that will optimally detect expected restriction maps (Figure 2); factors considered include the average size and distribution of restriction fragments produced after DNA methylation. Nominally, restriction maps constructed from individual molecules must present a sufficient density of restriction sites for confident map construction or alignment against a reference map. Since AluI DNA methylase targets the cytosine in the sequence AGCT, we selected a restriction enzyme that partially overlaps with the AluI methylation sites. (If overlap is complete, no restriction maps will be created.) The cognate sequence of the restriction enzyme NheI (G^CTAGC) overlaps with AluI methylation sites at the sequences: A***GC***^***Me***^***TAGC ***and ***GCTAGC***^***Me***^T, and at these NheI sites we expect to observe no cleavage. Data acquired from sufficient numbers of randomly sheared *E. coli *DNA molecules allow redundant coverage of the entire genome, thereby assaying all overlapping sites of NheI/AluI methylase revealed as missing NheI cuts in the alignment of the NheI consensus optical map to the *in silico *sequence map.

### Detection of AluI methylation sites in the *E. coli *genome

We evaluated strategy (*i*) across the entire *E. coli *genome using AluI methylase treated DNA, followed by optical mapping with NheI. AluI methylation modifies 13,335 sites in the *E. coli *genome, and it is expected to block cleavage at approximately 30% (51/158) of the NheI cleavage sites (see explanation provided below). A data set consisting of 1,377 NheI/AluI methylase *E. coli *optical maps (cleaved single molecules) was created (Methods). From the raw data set, we selected 631 maps larger than 550 kb as the most informative for the construction of the *de novo *optical map spanning the entire *E. coli *genome. This filtering process compensated for the relatively large, 42.70 kb, average fragment size observed in this data set by ensuring sufficient density of restriction cleavage sites across all considered molecules spanning the entire *E. coli *genome [24].

Given the experimentally derived average fragment size (42.70 kb), we formulated preliminary measures of DNA methylation. In the absence of AluI methylation, NheI optical maps are expected to have an average fragment size of 29.36 kb. Thus, the apparent digestion efficiency of methylated DNA was about 69%, so that ~30% of NheI cleavage sites were blocked by AluI methylation, or partially digested. The final assembled contig contained 176 optical maps indicating a 28% contig rate (Figure 3 panels B and C); the modest contig rate was due to the large average fragment size [24] of the map data set. Contig rate is calculated by dividing the number of maps in a contig by the total number of maps submitted for contig construction (176/631 × 100% = 28% for this data set).

Following assembly, the *de novo *optical map contig was aligned with the *in silico *map based on the *E. coli *sequence with the assumption of no methylation (Figure 3 panel D; subset of alignment shown). Based on this alignment, we were able to confidently identify 43 NheI sites as missing cuts blocked by AluI methylation (see Additional file 1). Given the known nucleotide locations of AluI methylation and NheI cleavage, we had expected 51 NheI sites to be blocked by AluI methylation. Of the 8 sites that were not readily identified, all but one represented small restriction fragments (2 kb and less). Three of the above instances showed irregularly sized fragments in the alignment. Of the 5 fragments not detected, 1 was not detected due to an assembly error, 3 were too small to be detected (~150 bp), and 1 small fragment (1.7 kb) was in a low coverage area where detection was not possible. Of course given more coverage, some of the above problems can be ameliorated.

### Dcm methylation profiling of *E. coli *strain K-12 MG1655

**Figure 4:**
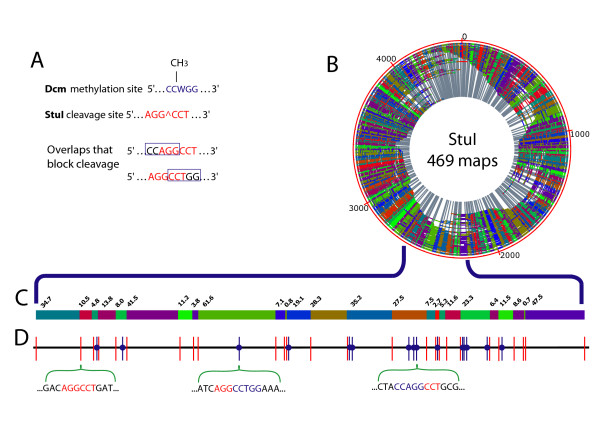
**Discernment of an *in vivo *methylation profile in *E. coli***. A; Overlap between sites of Dcm methylation and StuI cleavage. Dcm methylation and StuI cleavage sites are shown on top. The two overlaps between Dcm methylation (blue box) and StuI cleavage (red text) both block cleavage. B; The *de novo *optical map contig of MG1655 dcm^+ ^strain of *E. coli*, assembled from 469 StuI maps. Outer red circle indicates the size of the contigs, individual restriction fragments are denoted with alternating colors and grey lines. The origin of the optical map does not coincide with the start of the published sequence, because the optical map was assembled *de novo*. C; A representative section (~430 kb) of the *de novo *StuI optical map contig of dcm^+ ^*E. coli*. Alternating color blocks represent individual restriction fragments with their respective sizes (in kb) marked above the blocks. D; Comparison of in silico StuI map to *de novo *(C) Dcm^+ ^*E. coli *optical map contig. StuI cleavage sites are represented as vertical bars. Red bars indicate cleavage sites observed in the *de novo *map. StuI sites overlapping with Dcm methylation (absent in the optical map) are shown as a blue vertical bar with a blue circle. Below are two examples of local sequence showing overlap between DNA cleavage and methylation sites and one example of local sequence showing no overlap.

Given the positive results of genomic methylation profiling reported above on a synthetically methylated *E. coli *genome, the same methylation profiling strategy (*i*) was chosen to evaluate an *E. coli *strain bearing endogenous methylation sites. For this experiment, we optically mapped the *E. coli *strain K-12 MG1655, positive for Dcm methylase [38], which is a modification system methylating the internal cytosine in the recognition sequence CCWGG, where W is A or T. There are a total of 12,042 Dcm sites in the genome, and a portion of these sites are detected using the methylation sensitive restriction enzyme StuI, which cleaves AGG^CCT in *E. coli *at an average frequency of 7.66 kb. The two possible sites of Dcm StuI overlap that would block StuI cleavage include CC^***Me***^***AGGCCT ***and ***AGGCC***^Me^***T***GG (Figure 4 panel A).

A data set of 6,637 StuI optical maps was created for determining the Dcm methylation/StuI profile of *E. coli *K-12 MG1655. These optical maps were then pairwise aligned to the StuI *in silico *map of *E. coli *allowing selection of the 700 top scoring molecules for assembly (see Methods). This filtering step reduced the computational complexity and accelerated map assembly when using data sets boasting densely spaced restriction sites on mapped molecules. The final contig comprises 469 maps (Figure 4 panel B), indicating a 67% contig rate and an average fragment size of 11.75 kb.

The StuI (Dcm) contig map was aligned to the *in silico *StuI map using a map assembler [39,40]. We identified 128 StuI restriction sites as being methylated, assuming blockage from overlapping and adjacent methylation, by scoring a missing cut (in relation to the *in silico *map) in the optical contig map. Following the process used for the characterization of the methylation status of NheI restriction sites, we compared our experimentally determined StuI methylation profile to *in silico *sequence prediction. The *E. coli *genome contains 606 StuI cleavage sites, and 138 of these are expected to be blocked by Dcm methylation. Our methylation profiling results are in close agreement with this analysis. All but 10 sites were identified (128/138), and the 10 sites that were not all consist of small (< 2 kb), poorly detected, restriction fragments (see Additional file 2). One site was later identified as a mass increase; thus the number of undetected sites is 9.

### Exploring the human methylome

**Figure 5:**
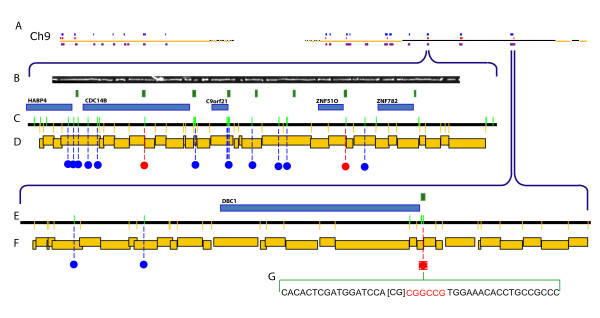
**Profiling methylation sites in the human genome**. Optical mapping tabulations of hyper- and hypomethylation across human Ch 9. A; Optical mapping findings of hypomethylation (16 – red marks) and hypermethylation (75 – blue marks) are shown aligned to an *in silico *SwaI restriction map (gold and black horizontal lines) of entire human Ch 9 (Build 35, hg17; 140 Mb). Optical maps constructed from a dual SwaI, EagI digestion and then overlapped forming contigs (purple boxes) are shown aligned to the *in silico *SwaI (methylation insensitive) map. B; Image of a single human DNA molecule (~400 kb) contained in the contig (469 kb) depicted in D; C, E; Detailed EagI (green vertical lines on track), SwaI (yellow vertical lines below line) *in silico *map of respective regions of human genome with blue (hypermethylation) and red (hypomethylation) dots showing methylation sites identified by optical mapping. Blue boxes represent genes, and green boxes show CpG islands. D, F; EagI, SwaI optical map contig with the restriction fragments size scaled and represented by staggered gold boxes. Contig D and F respectively span chromosome 9: Build 35, hg 17; start 96,297,748 bp, end 96,766,284 bp (D); start 118,802,475 bp, end 119,384,765 bp (F). G; An expanded view of a methylation call adjacent with Illumina findings showing nucleotide composition; red nucleotides show a hypomethylated EagI site with surrounding sequence (black). The CpG dinucleotide reported as hypomethylated by Illumina is bracketed [CG].

We then evaluated the methylation detection capabilities of optical mapping within the human genome, using strategy (*ii*) requiring one restriction enzyme for barcoding and a second for revealing methylation patterns. A set of optical maps was generated for the human embryonic stem cell line H1 (passage 44), using the restriction enzymes SwaI (ATTTÂAAT) for barcoding and EagI (C^CGCCG) for detection of methylated restriction sites (Figure 5). SwaI (barcoding) is an intrinsically C^Me^pG methylation insensitive restriction enzyme cleaving the human genome at an average frequency of ~15 kb, while EagI action is affected by methylation. In the absence of methylation, EagI cleaves human DNA producing restriction fragments with an average size of 32 kb. Not surprisingly given its CG-rich cognate sequence, EagI targets CpG islands, often multiply cleaving small CpG islands producing sub-microscopic restriction fragments. It is of significant interest that EagI targets about 78% of the 27,437 CpG islands (21,287/27,437). EagI cleaves the human genome at 89,473 sites (assuming no methylation). About half of the CpG islands (14,008/27,437) contain single EagI cuts, while another 7,279 of these targeted islands comprise multiple cleavage sites (CpG island statistics taken from Santa Cruz genome browser).

Ideally, the two-enzyme optical maps would be assembled into a genome-wide restriction map from which the methylation pattern of the genome would be inferred. Because our assembly algorithm does not support two-enzyme maps, we employed the following scheme. Briefly, two-enzyme optical maps are aligned to *in silico *two-enzyme maps of the human genome; maps that align are then stripped from the scaffold and independently assembled into contigs. The consensus maps from such contigs are then aligned back to *in silico *map (reference map) (Figure 5, panel A). This alignment supports elucidation of methylation patterns, in ways paralleling strategy (*i*), from comparison of experimentally derived EagI restriction sites (consensus maps) against the human reference map.

Given the above synopsis of our analysis scheme, EagI and SwaI cleavage are conveniently merged together, both during sequential digestion (Methods) and for methylation profiling, since pervasive CpG methylation greatly attenuates the number of cleavable EagI restriction sites. It is important to note that this tact enables confident placement of serially digested molecules (SwaI/EagI), using just SwaI restriction patterns on the human reference map. As such, our analysis readily considers cleaved EagI sites as "extra cuts," or modeled errors [41], appearing within a SwaI restriction map after comparison against the human reference map. Consequently, EagI restriction sites, reflecting lack of methylation, are identified by their intersection with an EagI (*in silico*) human reference map, which is overlaid upon the SwaI resource. Based on these analysis guidelines, we developed several optical map alignment approaches, described below, for building map data sets supporting methylation profiling of the human genome.

Optical maps, using a combination of SwaI and EagI digestion, are aligned to the human reference map using three complementary approaches (Methods): **(a) **pairwise alignment against an *in silico *SwaI map for capturing maps from heavily methylated regions with few EagI cuts; **(b) **pairwise alignment against the SwaI map with relaxed penalties for extra cuts (errors) for capturing maps from unmethylated loci; and **(c) **alignment against a SwaI/EagI *in silico *reference map for capturing maps from those genomic regions with below average densities of SwaI sites, but densely populated by available EagI sites.

We chose chromosome 9 for detailed analysis (Figure 5). Our data set contained 731 optical maps that aligned to chromosome 9 using at least one of the above approaches (a, b, and/or c). Following assembly, the molecules formed 30 contigs, of which 21 aligned back to *in silico *reference map. The 21 aligned contigs were assembled from 54 molecules and spanned 13.348 Mb. The genomic regions covered by these contigs contained 244 EagI sites. From the analysis of cleaved *vs*. uncleaved SwaI restriction sites tabulated on the set of aligned optical maps, we estimated the digest rate for SwaI as being about 85%. Since these very same DNA molecules were also cleaved with EagI under similar conditions, we reasoned that the EagI and SwaI digest rates were closely linked. Accordingly, estimation of EagI digestion rate allows confident assessment of methylation status using the analysis described below.

Since extra cut errors are random events and are only modestly observed in optical maps, these characteristics are leveraged for revealing unmethylated EagI restriction sites within contigs. Consider that the probability of an extra cut error occurring within a given interval of a mapped molecule is 1-e^-⌊*x*^; where ⌊ is the rate of extra cuts per Mb (usually estimated to be 3) and x is the interval in kb [34,35]. Using this analysis we identified 15 extra cuts (0.01 < p < 0.00001) in the consensus maps within 2 kb of an EagI site. These unmethylated *loci *contained 27 EagI sites (see Additional file 3) and all except 1 were located within CpG islands.

Since missing cut errors that are due to partial digestion are prevalent in optical maps, identification of methylated sites is more complex, and we deal with this issue by the development of analysis leveraging the clustered cleavage pattern shown by EagI in CpG islands. Accordingly, when several EagI cuts are in close proximity to each other, detection of methylation status in such clusters is actually enhanced because there are multiple opportunities for scoring cleavage events bounded by the spatial resolution of light microscopy. Consider that the maximum resolution of light microscopy corresponds to ~600 bp of DNA (~0.2 μm; fully stretched), so that a cluster of EagI cuts within a 600 bp region is imaged as one merged cleavage event. (Of course, this detection advantage also obscures the methylation status of closely spaced CpGs. Also, molecules are typically stretched to about 80% of their polymer contour length, and tiny restriction fragments tend to desorb from the surface, so that "merged" cleavage events include a greater span of about 2 kb.) Given an 85% digest rate, the probability of having no cuts within a cluster of n EagI cut sites is (1–0.85)^n^. We then identify methylated EagI sites (p < 0.0005) located in areas containing multiple EagI sites in close proximity (~2 kb) to each other that also do not show any corresponding cuts in the optical maps. In this way, we identified 12 such loci, containing a total of 55 EagI sites (Additional file 4). Essentially, this analysis allows confident calls by trading deep map coverage at a given genomic location for consideration of cleavage sites that are clustered.

The analysis of non-clustered (independent), methylated EagI sites follows a different strategy. Given the previously discussed SwaI and EagI digest rate of 85%, we designed data filters requiring a minimum depth of 2 molecules for calling methylation status; this analysis identified 68 methylated EagI sites (p < 0.0225) (see Additional file 4), with 6 sites located within CpG islands (Figure 5, panels B through F). Within this region we found that 27 out of 150 characterized EagI sites were cleaved, inferring an apparent methylation rate of 80%. The above rate is in agreement with the estimated rate of CpG methylation in the human genome [13,42]. Unmethylated CpG dinucleotides are localized primarily to CpG islands with only ~35% of the islands being methylated in stem cells [43]. In our findings unmethylated loci are largely associated with CpG islands while the methylated loci are located outside of CpG islands (see Additional files 3 and 4).

To further validate our methods, we intersected data from a recent (bead) microarray survey of human stem cell methylation [43] with our findings. A locus common to both datasets was further confirmed by bisulfite PCR. For example, the promoter region of the DBC1 gene contains a CpG island with 3 EagI cut sites. This locus is represented by 3 probes on the Illumina methylation bead array [43]. Corresponding optical mapping data show that one of the EagI sites located in the CpG island associated with the DBC1 promoter is cleaved, indicating hypomethylation. The Illumina results in the DBC1 locus (Illumina probe DBC1 1179) report the hypomethylation (14% methylation level) of the CpG dinucleotide (ch 9: 119,211,696 bp; build 35, hg 17) that is directly adjacent to the cleaved EagI site at (ch 9: 119,211,697 bp) (Figure 5, panel G). Our analysis also reports the adjacent EagI site as being hypomethylated. We developed primers for bisulfite PCR analysis of this locus, followed by cloning and sequencing (Methods). The sequencing results confirmed that the EagI restriction enzyme site (119,211,697 bp) is unmethylated (11 of 11 clones) in the H1 p44 human embryonic stem cell genome (Table 1). These data also demonstrate that all C's in the DBC1 sequence shown in Table 1 are unmethylated.

**Table 1:** Bisulfite sequencing of the DBC1 locus

F primer	GTA GGG TGT GTT TAT GT			
R primer	AAA AAA CTC TTA CTT CAT TCT			
				
dbc	CCTGAGTGTT	TCTGGGGCGG	CAGGTGTTTC	CA**CGGCCG [**CG]
				
7f	TTTGAGTGTT	TTTGGGGTGG	TAGGTGTTTT	TATGGTTGTG
7r	TTTGAGTGTT	TTTGGGGTGG	TAGGTGTTTT	TATGGTTGTG
18f	TTTGAGTGTT	TTTGGGGTGG	TAGGTGTTTT	TATGGTTGTG
18r	TTTGAGTGTT	TTTGGGGTGG	TAGGTGTTTT	TATGGTTGTG
4f	TTTGAGTGTT	TTTGGGGTGG	TAGGTGTTTT	TATGGTTGTG
4r	TTTGAGTGTT	TTTGGGGTGG	TAGGTGTTTT	TATGGTTGTG
12f	TTTGAGTGTT	TTTGGGGTGG	TAGGTGTTTT	TATGGTTGTG
12r	TTTGAGTGTT	TTTGGGGTGG	TAGGTGTTTT	TATGGTTGTG
3f	TTTGAGTGTT	TTTGGGGTGG	TAGGTGTTTT	TATGGTTGTG
3r	TTTGAGTGTT	TTTGGGGTGG	TAGGTGTTTT	TATGGTTGTG
2f	TTTGAGTGTT	TTTGGGGTGG	TAGGTGTTTT	TATGGTTGTG
2r	TTTGAGTGTT	TTTGGGGTGG	TAGGTGTTTT	TATGGTTGTG
16f	TTTGAGTGTT	TTTGGGGTGG	TAGGTGTTTT	TATGGTTGTG
16r	TTTGAGTGTT	TTTGGGGTGG	TAGGTGTTTT	TATGGTTGTG
13f	TTTGAGTGTT	TTTGGGGTGG	TAGGTGTTTT	TATGGTTGTG
13r	TTTGAGTGTT	TTTGGGGTGG	TAGGTGTTTT	TATGGTTGTG
6f	TTTGAGTGTT	TTTGGGGTGG	TAGGTGTTTT	TATGGTTGTG
6r	TTTGAGTGTT	TTTGGGGTGG	TAGGTGTTTT	TATGGTTGTG
8f	TTTGAGTGTT	TTTGGGGTGG	TAGGTGTTTT	TATGGTTGTG
8r	TTTGAGTGTT	TTTGGGGTGG	TAGGTGTTTT	TATGGTTGTG
10f	TTTGAGTGTT	TTTGGGGTGG	TAGGTGTTTT	TATGGTTGTG
10r	TTTGAGTGTT	TTTGGGGTGG	TAGGTGTTTT	TATGGTTGTG
	.........	.........	.........	.........
diff	TTTGAGTGTT	TTTGGGGTGG	TAGGTGTTTT	TATGGTTGTG

Sequences (forward and complement of reverse directions) of 11 bisulfite PCR product clones, aligned to the reference (top sequence). The EagI site in the DBC1 promoter is in red, data point from Illumina is bracketed. Both are shown to be unmethylated in our sample.

## Discussion and conclusion

We conclude that direct analysis of single genomic DNA molecules is a viable means for genome wide, *de novo *methylation profiling, based on our analysis of optical mapping data from several *E. coli *systems, engineered and wild, and a partial map of the human genome. Although restriction endonucleases are simple, reliable reagents for discernment of methylation patterns, their use on a whole genome basis has been limited for lack of complementary analysis systems for fully exploiting the practical advantages they intrinsically represent–methylation status at cognate sites directly revealed by cleavage without the use of damaging chemical modification steps, or amplification. In this regard, the optical mapping system, based on the high-throughput analysis of ordered restriction maps, offers whole genome methylation profiling capabilities working from unmodified, unamplified genomic DNA molecules that directly pinpoint cleavage events across genomes. PCR amplification, however, does in theory allow the analysis of any genomic locus, but practical considerations–primer design and number of amplicons–often limit comprehensive analysis of entire genomes.

Furthermore, optical mapping readily profiles repeat-strewn regions of mammalian genomes posing formidable challenges for techniques using both amplification and hybridization steps. On the other hand, optical mapping-based profiling is limited by those methylation sites interrogated by a given restriction enzyme; however, we have shown here that judicious choice of enzymes (SwaI/EagI) ensures significant sampling of critical genomic elements, such as CpG islands, despite very modest coverage of the entire human genome by this data set. Additional map coverage and other enzyme pairs targeting additional genomic elements (*i.e*., LINES) would greatly augment the scope of our human methylation profiling approach. This scope is limited by the size of restriction fragments produced by a selected enzyme. Such limitations arise because small restriction fragments are not uniformly detected, so that their occurrence limits enzyme choice and spatial resolution of methylation patterns. However, if the algorithm used for the detection of DNA methylation presented in this paper is combined with map data using the recently published optical barcoding system [44] – using direct labeling in place of restriction digestion – the limitations imposed on the method by enzyme choice will be largely alleviated.

In many ways, the work we have presented here resembles classical "footprinting" approaches, where nuclease action is attenuated by the occurrence of protein-DNA complexes as assayed by gel electrophoresis. Instead, our findings show the footprint detection of modified DNA sites. As such, we envision genomic footprinting of transcription factors and other DNA binding proteins using the approaches we have presented, and those we will develop around the recently published DNA barcoding approach [44] using nicking restriction enzymes and fluorochrome labeling in place of the assessment of restriction fragments. This new approach would likely complement the capabilities of the Cognate Site Identifier [45] technique by use of genomic targets fully presenting native patterns of DNA modification and comprehensively addressable genomic repeats. Lastly, we also envision that mammalian genomes will be profiled by optical mapping for both methylation sites and structural variants (Copy Number Variants) [31] through analysis of deep single molecule data sets revealing altered patterns of genomic structure and DNA modification.

## Methods

### Bacterial culture strains and preparation of genomic DNA

*E. coli *genomic DNA agarose inserts [46] were prepared from a culture grown overnight in a shaker using LB media. To remove excess EDTA and null proteinase K activity, inserts were washed five times, the first time being overnight, in TE (10 mM Tris, 1 mM EDTA; pH 8.0) and supplemented with 1.0 mM phenylmethylsulfonyl fluoride (PMSF). Following wash steps, inserts were melted at 78°C for 5 minutes, and then treated with β-agarase (NEB; 110 μl TE + 1 unit of β-agarase per 20 μl of agarose) solution at 42°C for 4 hr.

### Methylation of genomic DNA

*E. coli *genomic DNA inserts that have been washed in TE were treated with 20 units of AluI methylase (NEB) in a total buffer volume of 200 μl (including the 80 μl insert) supplemented with 0.5 μl of NEB stock S-adenosyl-methionine (SAM) overnight at 37°C. The efficiency of the methylation reaction was tested with an "in-tube" restriction digest, followed by gel electrophoresis, showing that the cleavage activity of the AluI restriction enzyme was significantly inhibited (data not shown).

### Mammalian genomic DNA preparation

Human embryonic stem cell line H1 was cultured in a feeder cell independent media according to published protocol [47]. Upon reaching passage 44 cells were harvested and frozen in storage media (growth media supplemented with DMSO).

To prepare genomic DNA for optical mapping, aliquots of 1 × 10^6 ^cells were thawed on ice. Following thawing, cells were washed twice with PBS. Liquid lysates of genomic DNA were prepared by diluting hES cells in a solution of 0.1 M EDTA and 10 mM EGTA, pH 8.5, supplemented with 1 μg/ml of Proteinase K, at concentrations ranging from 10 to 200 cell/μl. Following dilution the lysates were heated to 50°C for 1 hr, and then incubated at 37°C overnight. Lysates were then stored at 4°C. Lysates containing 25–50 cell/μl yielded the best results.

### Bisulfite treatment of genomic DNA

Bisulfite conversion of human genomic DNA was performed using the EZ DNA methylation kit (Zymo Research, Orange CA [catalog # D5001]), according to manufacturer's instructions.

### Bisulfite PCR

50 ng of bisulfite converted genomic DNA was used per PCR reaction using DNA *taq *polymerase and buffers from the Expand Long Template PCR system (Roche Applied Science, Indianapolis IN (catalog # 11 681 834 001)). The following primers were used for amplification of the bisulfite treated DBC1 locus: forward primer GTA GGG TGT GTT TAT GT, reverse primer AAA AAA CTC TTA CTT CAT TCT. The primers were designed using the BiSearch Primer Design and Search Tool [48,49]. The following thermocycler program was used for amplification: 1 cycle 50°C, 2 min; 95°C, 12 min; and 40 cycles: 95°C, 20 sec; 56°C, 30 sec; 72°C, 1 min. For control amplifications of the DBC1 locus the following primers were used: forward primer TAT GCG CAC GAG CAT CCA, reverse primer TAC GTA GAG AAG CTC TTG CTT, with conditions of amplification being identical to the above.

### PCR product cloning

Bisulfite PCR products were cloned using a Strataclone™ PCR cloning kit (Stratagene, La Jolla CA (catalog # 240205)), according to the kit protocol. Colony screening was conducted using the colony PCR procedure, and clones containing the correct insert were sequenced at the UW Biotechnology Center DNA Sequencing Laboratory.

### Surface preparation

Glass cover slips (22 × 22 mm, Fisher's Finest, Fisher Scientific) were cleaned and derivatized according to previously published protocols [17].

### DNA mounting, overlay, digestion and staining

DNA molecules were mounted on derivatized glass surfaces via capillary action utilizing a microfluidic device [33]. To provide a sizing standard, bacteriophage DNA was co-mounted with genomic DNA. A thin layer of acrylamide (3.3% containing 0.02% Triton X-100 [Sigma]) was applied to each surface. Following application the acrylamide overlay was washed twice for 2 min with 400 μl of TE and once with 200 μl of digestion buffer for the same amount of time. The restriction digest was performed by adding to each surface 200 μl of restriction buffer (NEB buffer 2) supplemented with 20 units of either NheI (NEB) or StuI (NEB) restriction enzymes. The surfaces were then incubated for 2 hr at 37°C in a humidified chamber.

For two enzyme, human optical maps, surfaces were first treated for 2 hr at 25°C with 200 μl of restriction buffer (NEB buffer 3) containing 20 units of the restriction enzyme SwaI (NEB). The first mixture was aspirated off, and 200 μl of restriction buffer (NEB buffer 3) with 20 units of the restriction enzyme EagI (NEB) was added. The surface was then incubated in a 37°C humidity chamber for an additional 2 hr. Following digestion, the surfaces were washed twice with 500 μl of TE for 5 minutes. The surfaces were mounted onto a glass slide with 12 μl of 0.2 μM YOYO-1 solution (containing five parts YOYO-1 solution and 95 parts of β-mercaptoethanol in TE 20% v/v). The samples were sealed with nail polish and incubated in the dark for 20 min allowing the staining dye to diffuse.

### Image acquisition and processing

Surface mounted DNA samples were imaged in a fully automated fashion with a 63× objective (Zeiss) and a high resolution digital camera [23,33]. Co-mounted bacteriophage molecules were used to determine both the digest rate and to provide a sizing standard for integrated fluorescence intensity measurements [20]. Machine vision software (Pathfinder) was used to create optical maps from imaged molecules [32,33].

### Pairwise alignments of optical maps

Optical maps were aligned to the *in silico *maps using pairwise alignment. Optimal alignments were found using an implementation of the Smith-Waterman algorithm for restriction maps with a heuristic scoring function motivated by a likelihood ratio test for the distinguishing spurious alignments from optical mapping error [34,41]. An alignment of two maps is a pairing of the cut sites of the two maps. Each pair of cut sites is given a numerical score. This score is positive if the adjacent fragment lengths are of comparable length. The score is penalized as the lengths differ and also if the adjacent fragment pairs have cut site differences. The score of an alignment of two maps is the sum of the scores of their aligned pairs of cut sites. For two maps of lengths n and m respectively, 2 nm alignments are possible. The Smith-Waterman algorithm is an efficient method for finding that alignment with the highest score. By definition, any two maps will have a highest-scoring alignment. However, that alignment may be spurious and not biologically meaningful. Because there is no way to guarantee that an alignment is or is not spurious, one needs to use statistical methods for excluding spurious alignments from the assembly inputs.

### Optical map assembly

The optical map assembler was used to construct *de novo E. coli *consensus maps [19-21,39,40]. The assembler uses a dynamic programming algorithm to assemble individual optical maps into contigs. The assembler has built in error checking and correction features, as well as a number of user defined variables. For the AluI methylated *E. coli *NheI *de novo *optical map the assembly T value was 0.001; the false circular probability was set to 0.01. The final quality score reported as false circularization probability (FP) was 0.057. For the Dcm methylated StuI optical map assembly, a dynamic range of T values was used: 0.000001, 0.00001, 0.0001 and a false circular probability of 0.01 was used. The final quality score for the contig was FP = 0.015.

### Optical map contig (consensus) to *in silico *map alignment

The map assembler was used to align contig consensus optical maps to sequence derived *in silico *maps [39,40]. *De novo *maps were aligned to the *in silico *map with very stringent parameters in regards to sizing error and the probability of fragments missing in the *de novo *maps. In the resulting alignment, sites of DNA methylation in the *de novo *map appeared as missing cuts in reference to the *in silico *maps. The *in silico *map was used as a seed and the following map assembler parameters were used; a dynamic T value range 0.001; 0.01; 0.1, false circular probability of 0.1, the probability of missing 1 kb fragment was set to 0.

### Optical map alignment and assembly–two enzymes

Optical maps generated with two enzymes (SwaI and EagI) were pairwise aligned to an *in silico *map of the human genome. Optimal alignments were found using an implementation of the Smith Waterman algorithm for restriction maps with a heuristic scoring function motivated by a likelihood ratio test for the distinguishing spurious alignments from optical mapping error [34,41]. The following algorithms were used: (1) optical maps were aligned to a SwaI *in silico *map using our lab's default pairwise alignment parameters; (2) optical maps were aligned to a SwaI *in silico *map, with a lowered alignment penalty for extra cuts; (3) optical maps were aligned to a two enzyme (SwaI and EagI) *in silico *map of the human genome, with a lowered penalty for missing cuts. Optical maps that aligned using one of the above were pooled into one map set. The composite map set was used to produce contigs with map assembler. The resulting contigs were aligned to an *in silico *SwaI map of the human genome based on build 35 (hg17) [50].

## Authors' contributions

GEA – Performed all experiments, contributed to experimental strategies and drafted manuscript. SG – Performed the computational and statistical aspects of methylation detection and optical map assembly; also, contributed parts to the manuscript. RR – Wrote the image processing programs and software used for human restriction fragment detection. DKF – Contributed to writing the imaging software. SZ – Contributed to the development of the optical mapping system. KP – Contributed to the development of the optical mapping system. CPC – Created the database and Java tools used for optical mapping. VB – Cultured the human embryonic stem cells and contributed scientific ideas to hES DNA isolation. JAT – Provided expertise on human embryonic stem cell lines and biology. DCS – Conceived and supervised this work and co-wrote the manuscript.

## Supplementary Material

Additional file 1 ***E. coli *AluI methylation NheI cleavage**. A detailed spreadsheet describing the optical map of an engineered methylation profile in *E. coli*. Click here for file

Additional file 2 ***E. coli *Dcm methylation StuI**. A detailed spreadsheet describing the optical map of an endogenous methylation profile in *E. coli*. Click here for file

Additional file 3 **EagI cuts present in the OM data**. Statistical information about detecting hypomethylated regions in the human genome, *via *an optical map. Click here for file

Additional file 4 **EagI cuts not present in the OM Data**. Statistical information about detecting hypermethylated regions in the human genome, *via *an optical map. Click here for file
